# Indole-4-carboxaldehyde Isolated from Seaweed, *Sargassum thunbergii*, Attenuates Methylglyoxal-Induced Hepatic Inflammation

**DOI:** 10.3390/md17090486

**Published:** 2019-08-21

**Authors:** Seon-Heui Cha, Yongha Hwang, Soo-Jin Heo, Hee-Sook Jun

**Affiliations:** 1 Department of Marine Biomedical Sciences, Hanseo University, Chungcheongnam-do 31962, Korea; 2 Lee Gil Ya Cancer and Diabetes Institute, Gachon University, Incheon 21999, Korea; 3 College of Pharmacy, Gachon University, Incheon 21999, Korea; 4 Jeju International Marine Science Center for Research & Education, Korea Institute of Ocean Science & Technology (KIOST), Jeju 63349, Korea; 5 Gachon Medical and Convergence Institute, Gachon Gil Medical Center, Incheon 21999, Korea

**Keywords:** hepatic steatosis, metabolic disease, AGEs, seaweed, *Sargassum thunbergii*

## Abstract

Glucose degradation is aberrantly increased in hyperglycemia, which causes various harmful effects on the liver. Glyoxalase-1 (Glo-1) is a ubiquitous cellular enzyme that participates in the detoxification of methylglyoxal (MGO), a cytotoxic byproduct of glycolysis that induces protein modification (advanced glycation end-products, AGEs) and inflammation. Here, we investigated the anti-inflammatory effect of indole-4-carboxaldehyde (ST-I4C), which was isolated from the edible seaweed *Sargassum thunbergii*, on MGO-induced inflammation in HepG2 cells, a human hepatocyte cell line. ST-I4C attenuated the MGO-induced expression of inflammatory-related genes, such as tumor necrosis factor (TNF)-α and IFN-γ by activating nuclear factor-kappa B (NF-κB) without toxicity in HepG2 cells. In addition, ST-I4C reduced the MGO-induced AGE formation and the expression of the receptor for AGE (RAGE). Interestingly, both the mRNA and protein expression levels of Glo-1 increased following ST-I4C treatment, and the decrease in Glo-1 mRNA expression caused by MGO exposure was rescued by ST-I4C pretreatment. These results suggest that ST-I4C shows anti-inflammatory activity against MGO-induced inflammation in human hepatocytes by preventing an increase in the pro-inflammatory gene expression and AGE formation. Therefore, it represents a potential therapeutic agent for the prevention of hepatic steatosis.

## 1. Introduction

Methylglyoxal (MGO), a dicarbonyl compound, is a ubiquitous product of cellular metabolism and is therefore present in all cells, under both normal and pathological conditions. MGO can be produced as a by-product of protein and fatty acid metabolism [1,2], and the glycolytic pathway represents the most important endogenous source of MGO [3]. MGO is one of the most potent glycating agents; thus, MGO readily reacts with lipids, nucleic acids, and lysine and arginine residues of proteins to form advanced glycation end-products (AGEs) [4,5]. In addition to the direct changes in protein function caused by MGO modifications, AGE-modified proteins also exert cellular effects through their interaction with the specific AGE receptor, RAGE (receptor for AGE) [6,7], which triggers an inflammatory response at the cellular level and accounts for AGE toxicity. AGEs play a critical role in various pathophysiological mechanisms, including those associated with diabetic complications [8] and non-alcoholic fatty liver disease (NAFLD) [9,10]. 

MGO is produced during glucose utilization, and the levels of MGO increase under hyperglycemic conditions [11,12]. Increased plasma levels of MGO have been implicated in various metabolic diseases such as obesity and fatty liver disease, including NAFLD [13,14]. The concentration of MGO is elevated under high-glucose conditions, such as diabetes [15], and the MGO concentration increases in response to fructose stress in hepatocytes [16], consequently causing AGE formation and inflammation in the liver [17]. 

The brown seaweed *Sargassum thunbergii* is a common intertidal seaweed species found along the northwestern Pacific coast, which is characterized by a warm-temperate and humid maritime climate [18]. This seaweed is commonly used as bait and a component of artificial *Sargassum* beds because of its wide ecological amplitude and high economic and ecological value [18,19]. Crude extracts from *S. thunbergii* have been shown to have neuroprotective and antioxidant activities [20], a quinone derivate from this species exhibited anti-adipogenic and pro-osteoblastogenic activities [21], and indole derivates from the species were found to inhibit adipogenesis [22]. 

Indole-4-carboxaldehyde (ST-I4C) is a component isolated from an edible seaweed, *S. thunbergii*. There have been few studies on the health benefits of ST-I4C. A study has shown that ST-I4C exerted anti-adipogenic activity [22]. To date, no study has examined the anti-inflammatory effect of ST-I4C on MGO-induced inflammation or its inhibitory effect on MGO-induced AGE production. Therefore, the aim of this study was to determine whether ST-I4C has an anti-inflammatory effect on MGO in consideration of the importance of hepatic function in metabolic diseases. 

## 2. Results

### 2.1. ST-I4C Prevents MGO-Induced Inflammatory-Related Gene Expression in HepG2 Cells

**Figure 1:**
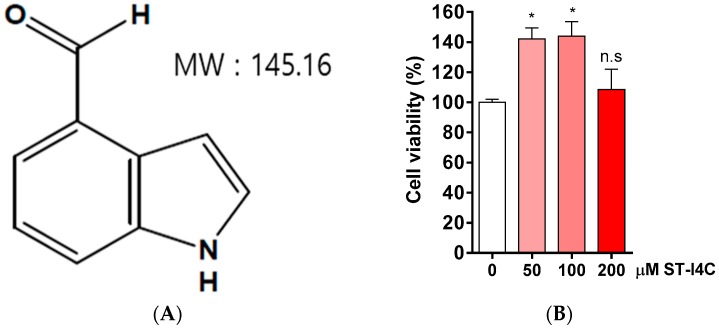

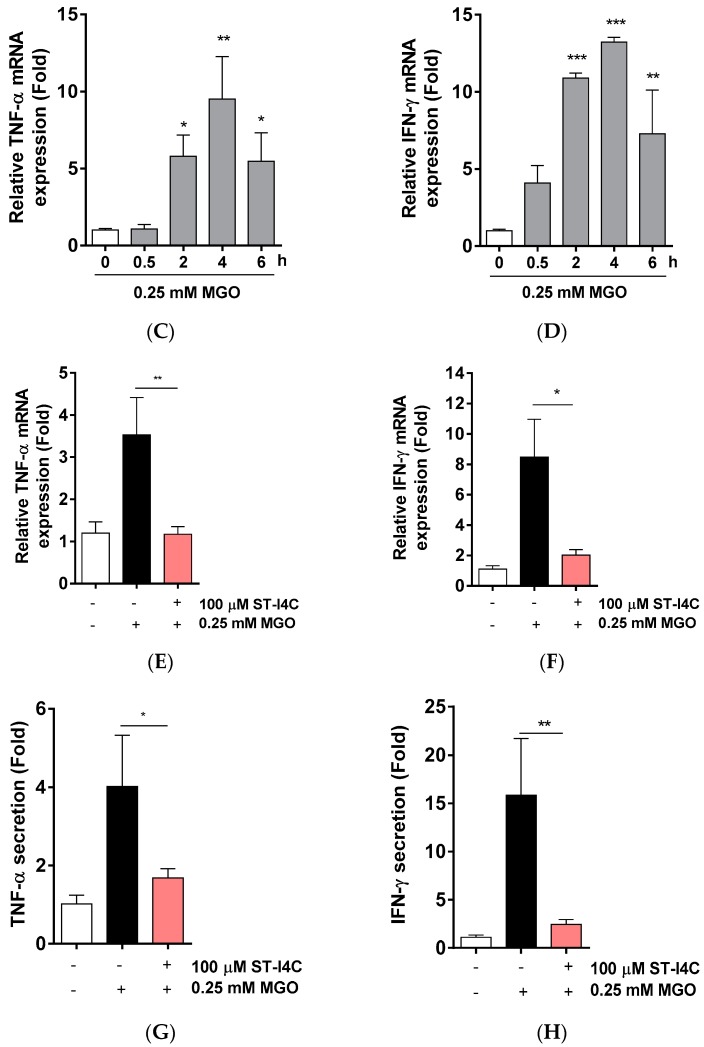
Indole-4-carboxaldehyde (ST-I4C) prevented methylglyoxal (MGO)-induced inflammatory-related mRNA expression in HepG2 cells. (**A**) Chemical structure of indole-4-carboxaldehyde (ST-I4C). (**B**) HepG2 cells were incubated with the indicated concentrations of ST-I4C for 24 h. Cell viability was measured by CCk-8 assay. (**C**,**D**) HepG2 cells were incubated with 0.25 mM MGO for the indicated times. (**C**) Tumor necrosis factor (TNF)-α and (**D**) IFN-γ mRNA expression was measured by qRT-PCR. (**E**,**F**) HepG2 cells were incubated with or without 100 µM ST-I4C for 2 h, and then further incubated with 0.25 mM MGO for 4 h. (**E**) TNF-α and (**F**) INF-γ mRNA expression was measured by qRT-PCR. Secreted cytokine of (**G**) TNF-α and (**H**) INF-γ was measured by enzyme-linked immunosorbent assay (ELISA). Experiments were performed in triplicate. * *p* < 0.05, ** *p* < 0.01, *** *p* < 0.001, n.s. indicates no significance.

Non-alcoholic fatty liver diseases (NAFLD) is associated with inflammation [23], so we determined whether ST-I4C (Figure 1A) attenuates MGO-induced inflammation. We first examined the cytotoxicity of ST-I4C. The results indicated that ST-I4C induced HepG2 cell proliferation at the tested concentration of 50 and 100 µM, but ST-I4C at 200 µM did not increase cell proliferation in HepG2 cells (Figure 1B). Next, to determine whether MGO induces inflammation in HepG2 cells, the cells were treated with 0.25 mM MGO for 0.5, 2, 4, and 6 h. The results indicated that the mRNA expression of pro-inflammatory cytokines, including tumor necrosis factor (TNF)-α and IFN-γ, increased in a time-dependent manner through 4 h (Figure 1C,D). On the other hand, other inflammatory cytokines were not change by 0.25 mM MGO exposure (Figure S1). We then used a 4 h incubation time for the following experiment. Next, to determine whether ST-I4C reduces MGO-induced TNF-α and IFN-γ mRNA expression, the cells were incubated with 100 µM ST-I4C for 2 h prior to 0.25 mM MGO treatment. As expected, the increased expression of both mRNAs was reduced by ST-I4C pretreatment (Figure 1E,F), indicating that ST-I4C may protect against inflammation. 

### 2.2. ST-I4C Inhibits MGO-Induced Activation of Nuclear Factor-Kappa B (NF-κB) 

**Figure 2:**
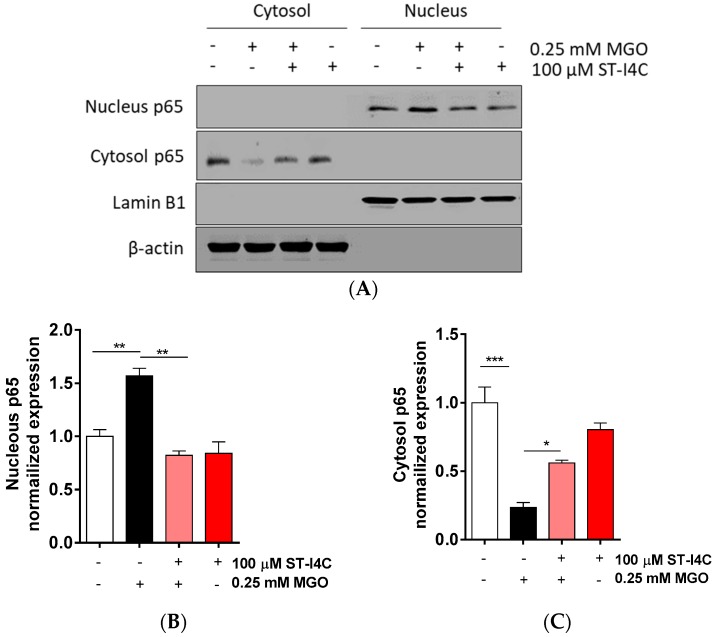
ST-I4C inhibited MGO-induced NF-κB activation. HepG2 cells were incubated with or without 100 μM ST-I4C for 2 h and then further incubated with or without 0.25 mM MGO for 4 h. Nuclear and cytoplasmic fractions were prepared, and Western blotting was subsequently performed. (**A**) The result of Western blot, (**B**,**C**) the bar graph were quantified from (A). Experiments were performed in triplicate. * *p* < 0.05, ** *p* < 0.01, *** *p* < 0.001.

After finding that ST-I4C prevented the MGO-induced overproduction of pro-inflammatory cytokines, including TNF-α and IFN-γ, we aimed to determine how ST-I4C regulates inflammation-related cytokine expression. The activation of the transcription factor NF-κB plays a central role in the inflammatory response [24]. Thus, we determined whether ST-I4C inhibits NF-κB activation in response to MGO. The results indicated that NF-κB translocation into the nucleus was induced by exposure to MGO, and its translocation was prevented by ST-I4C treatment prior to MGO exposure (Figure 2), suggesting that ST-I4C may inhibit MGO-induced NF-κB activation. 

### 2.3. ST-I4C Reduces AGEs Formation and RAGE mRNA Expression Levels Following MGO Exposure in HepG2 Cells 

**Figure 3:**
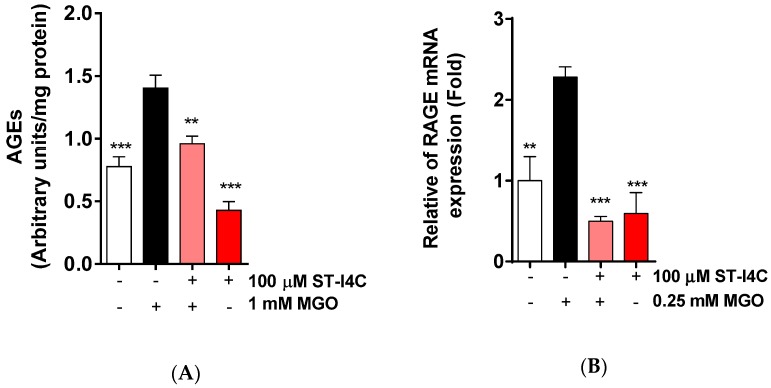
ST-I4C reduced MGO-induced advanced glycation end-product (AGE) formation and the RAGE mRNA expression level. (**A**) HepG2 cells were incubated with or without 100 µM ST-I4C for 2 h and then further incubated with 1 mM MGO for 24 h, after which the AGE content was measured. (**B**) HepG2 cells were incubated with or without 100 µM ST-I4C for 2 h and then further incubated with 0.25 mM MGO for 4 h, and RAGE mRNA expression was measured by qRT-PCR. Experiments were performed in triplicate. ** *p* < 0.01, *** *p* < 0.001.

MGO is a precursor of AGEs, so we determined whether ST-I4C reduces AGE formation in response to MGO exposure. AGE formation was increased by MGO exposure, but this increase in AGE formation was decreased to a level similar to that in the control by ST-I4C pretreatment (Figure 3A). Moreover, MGO exposure induced the mRNA expression of RAGE (receptor for AGE), and ST-I4C pretreatment prior to MGO exposure inhibited this increase (Figure 3B), suggesting that ST-I4C may modulate AGE formation. 

### 2.4. ST-I4C Induces Glyoxalase-1 Expression in HepG2 Cells 

**Figure 4:**
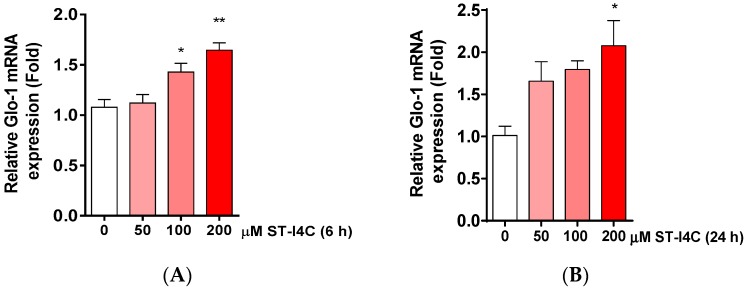

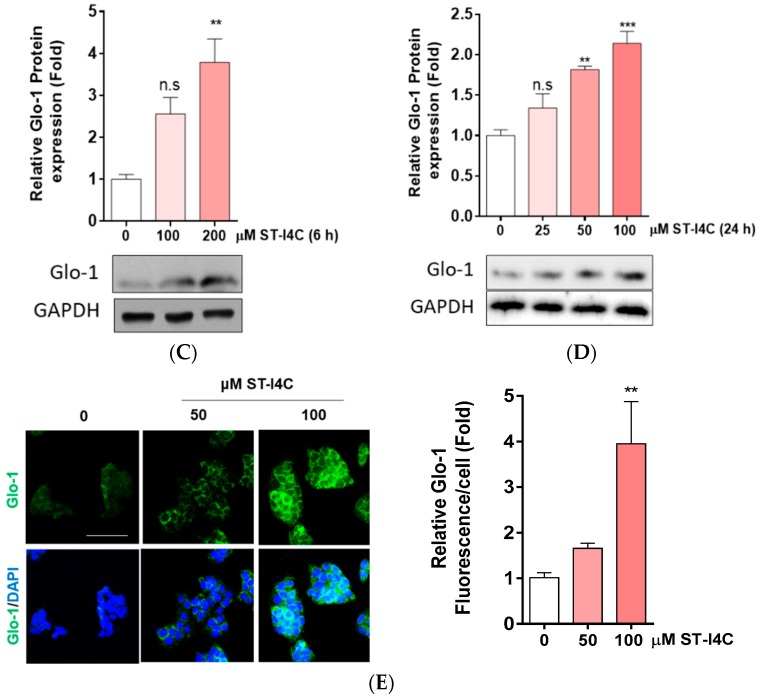
ST-I4C induced glyoxalase-1 (Glo-1) mRNA and protein expression level in HepG2 cells. HepG2 cells were incubated with the indicated concentrations of ST-I4C for 6 h (**A**,**C**) and 24 h (**B**,**D**,**E**). (**A**,**B**) Glo-1 mRNA was measured by qRT-PCR, and the protein expression of Glo-1 was measured by (**C**,**D**) Western blotting and (**E**) confocal microscopy. Scale bar: 50 µm. Experiments were performed in triplicate. * *p* < 0.05, ** *p* < 0.01, *** *p* < 0.001, n.s. indicates no significance.

It has been reported that an increase in the Glo-1 expression level decreases AGEs formation [25]. Glo-1 is a ubiquitous cellular enzyme in the glyoxalase system that participates in the detoxification of MGO, a cytotoxic byproduct of glycolysis [26]; Glo-1 is also used as a biomarker for NAFLD [27]. Thus, we examined the Glo-1 mRNA and protein expression levels and found that the mRNA expression of Glo-1 was significantly increased by ST-I4C treatment in a dose-dependent manner (Figure 4A). In addition, protein expression was induced by ST-I4C treatment (Figure 4B). To confirm these results, cells were stained with Glo-1 antibody after ST-14C treatment, and Glo-1 expression was increased by ST-I4C in a dose-dependent manner (Figure 4C). These results suggest that ST-I4C may induce the expression of Glo-1. 

### 2.5. Glo-1 Knockdown Abolished Anti-Inflammatory and Anti-Glycation Effects of ST-I4C in HepG2 Cells

**Figure 5:**
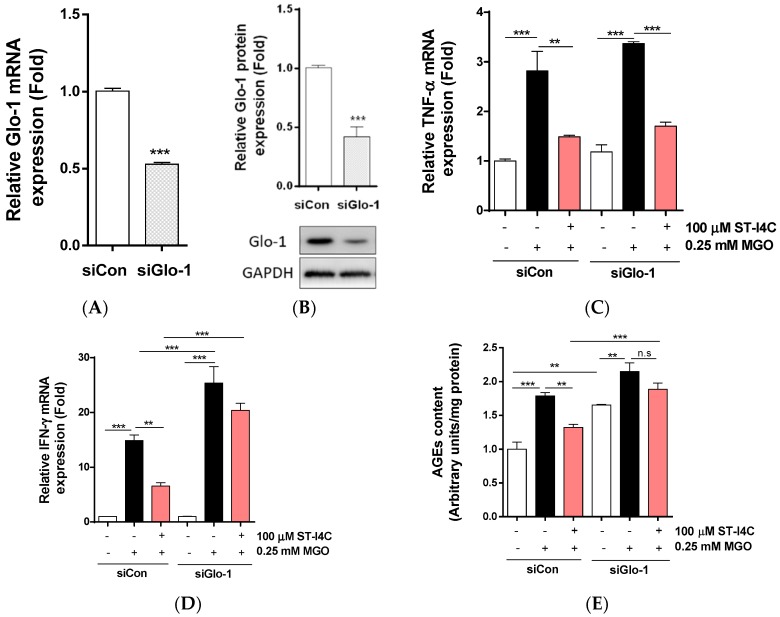
ST-I4C did not inhibit the mRNA expression of pro-inflammatory cytokines and formation of AGEs in Glo-1 knockdown HepG2 cells. HepG2 cells were transfected with Glo-1 siRNA for 24 h. Glo-1 (**A**) mRNA and (**B**) protein expression; the cells were incubated with or without 100 µM ST-I4C Figure 2. h and then further incubated with 0.25 mM MGO for 4 h. mRNA expression of (**C**) TNF-α and (**D**) IFN-γ was measured by qRT-PCR. (**E**) AGE formation was measured. Experiments were performed in triplicate. ** *p* < 0.01, *** *p* < 0.001.

ST-I4C induces the expression of Glo-1 mRNA and protein and reduces pro-inflammatory cytokines in response to MGO exposure. Thus, we determined whether the downregulation of pro-inflammatory cytokine expression by ST-I4C is mediated via Glo-1. Glo-1 knockdown using siRNA was confirmed by checking mRNA and protein expression levels. Glo-1 expression was reduced to about 50% of the control value at the mRNA level (Figure 5A) and to about 40% of the control value at the protein level (Figure 5B). The mRNA expression of TNF-α and IFN-γ was induced by MGO exposure, whereas the increase in the mRNA expression of these cytokines was abolished by ST-I4C treatment in the control siRNA transfected cells. Interestingly, MGO-induced TNF-α mRNA expression was not different between the control and Glo-1 siRNA transfected cells (Figure 5C), and the increase in IFN-γ mRNA expression induced by MGO exposure was higher in Glo-1 siRNA transfected cells than in the control siRNA transfected cells; this increase was not reduced by ST-I4C treatment prior to MGO exposure (Figure 5D). AGE formation in untreated cells was significantly higher in Glo-1 siRNA transfected cells than in the control siRNA transfected cells. In addition, MGO exposure further increased AGE formation in the Glo-1 siRNA transfected cells. MGO-induced AGE formation was significantly reduced by ST-I4C pretreated in the control siRNA transfected cells; however, this effect was not observed in the Glo-1 siRNA transfected cells (Figure 5E). These results suggest that the protection by ST-I4C against inflammation may be mediated through Glo-1. 

## 3. Discussion

Hepatic inflammation is one of major symptoms of NAFLD [28]. In the current study, we found that MGO-induced increases in pro-inflammatory cytokine production, AGE content, and RAGE expression were attenuated by ST-I4C pretreatment prior to MGO exposure in HepG2 cells. Some reports on indole derivatives isolated from natural sources, including seaweed, have indicated that they have various biological activities [29,30]. However, there is currently a lack of data on the effect of indole derivatives on hepatic steatosis caused by MGO, a highly reactive carbonyl and AGE precursor. Thus, in the present study, we isolated the indole derivative indole-4-carboxaldehyde (ST-I4C) from *S. thunbergii* and investigated its potential inhibitory effect on inflammation and Glo-1 induction in HepG2 cells. 

NAFLD is increasingly becoming the most common chronic liver disease, and is closely associated with obesity, type 2 diabetes, insulin resistance, hypertension, dyslipidemia, hypercholesterolemia, and a pro-inflammatory state [31,32]. Obesity is often associated with insulin resistance and represents a chronic low-grade inflammatory state, which is characterized by elevated circulating levels of cytokines and activation of pro-inflammatory signaling pathways [33]. Moreover, several studies have reported that there is a link between inflammation and NAFLD [23,34,35]. Several studies focused on the role of numerous inflammatory mediators such as tumor necrosis factor (TNF)-α, interleukin-6 (IL-6), IL-10, and adiponectin are considered to be the major inflammatory mediators found in NAFLD [36]. Inflammatory cytokines such as tumor necrosis factor (TNF)-α and interleukin-6 (IL-6) are considered to be the major inflammatory mediators in NAFLD [36]. Treatment with anti-TNF antibody and metformin, an antidiabetic drug that inhibits hepatic TNF-α expression, was found to improve steatosis in *ob*/*ob* mice [37,38]. Thus, hepatic inflammation was attenuated in IFN-γ deficient mice [39]. In accordance with these results, ST-I4C attenuated the mRNA expression of pro-inflammatory cytokines, particularly TNF-α and IFN-γ specific (Figure 1G,H; Supplementary Figure S1), in the present study. 

The link between inflammation and NAFLD has been well established; however, its molecular pathogenic mechanisms have not been completely characterized. Glo-1 is a critical cytosolic enzyme involved in the detoxification of reactive dicarbonyls, principally MGO. MGO is a major source of AGEs, and AGEs induce the sustained activation of AGE receptors (RAGE). The disruption of the glyoxalase system and the buildup of MGO are thought to be key factors in diabetes and its complications [13]. Several studies have shown that Glo-1 expression in the liver is decreased in response to both high-fructose and high-fat diets [39,40,41], and increased levels of AGEs in the liver have been associated with steatosis severity in patients with NAFLD [42]. Despite these associations, the specific role of Glo-1 in the pathogenesis of NAFLD is unknown. Glo-1 has also been implicated in obesity-related conditions [43]. In addition, TNF-α was shown to induce the phosphorylation of Glo-1, which induces cell death [44], and to affect the formation of MGO-derived AGEs [45]. Moreover, RAGE acts as a mediator in NF-κB pathway activation [46]. Therefore, much attention has been focused on identifying Glo-1 inducers in natural products [47,48,49]. 

Natural compounds, including seaweeds, have frequently been studied in models of NAFLD and seem to display beneficial effects. Initially, the health benefits of natural products were attributed to their potent antioxidant activity, and further research revealed that other activities, such as anti-inflammatory and metabolic effects, also contributed. Some natural products actually display a multitude of activities, and therefore, some natural constituents are considered not just antioxidants but bioactive compounds [50]. A bioactive compound produces a biological response via an array of subtle effects via different targets [51,52]. This multifarious mode of action of natural products seems appropriate for use in the treatment of NAFLD [53], in which various pathways are involved. Interestingly, molecules containing indole have been identified as promising drugs for the treatment of inflammation [54]. In addition, some synthetic indole compounds attenuate inflammation [55] by inhibiting cyclooxygenase (COX), which is the key enzyme in the biosynthesis of inflammation mediators, prostaglandins. 

There are many steps in AGE formation. Therefore, antiglycation effects may occur at any step such as reducing free radical production, blocking the carbonyl or dicarbonyl groups, inducing the glyoxalase system, inhibiting the generation of Amadori products in the advanced phase, blocking the crosslinking of AGE, chelating metal ions, blocking the function of RAGE, and reducing the subsequent development of oxidative stress and inflammation [55]. In this study, we showed that MGO exposure induced the mRNA expression of RAGE and that its elevation was reduced by pretreatment with ST-I4C by inducing Glo-1 mRNA and protein expression levels. 

RAGE mediates the binding and internalization of AGEs. RAGE is thought to initiate oxidative stress [54], which has been shown to cause cellular damage [55]. The cellular damage caused by AGEs is thought to result from the activation of NF-κB, which leads to the upregulation of cytokines [56]. In this study, MGO exposure caused an increase in AGE receptors, which resulted in the activation of NF-κB and led to an increase in inflammatory cytokine expression, which was reduced by ST-I4C. Although ST-I4C significantly decreased the MGO-induced increase in inflammatory cytokines, including TNF-α, and decreased AGE formation, Glo-1 siRNA transfection did not change the mRNA expression of TNF-α. It is speculated that the regulation of TNF-α by ST-I4C is possibly mediated through a variety of mechanisms because RAGE can bind many ligands, including AGEs, high-mobility group box protein 1, β-amyloid, and even lipopolysaccharide [57,58]. In a previous study, RAGE was found to be elevated in the liver in diabetic conditions, which not only favors glycation by reducing AGEs but also induces inflammation by reacting with other ligands [59,60,61]. 

Accordingly, ST-I4C may reduce AGE accumulation and RAGE expression to improve hepatic steatosis by increasing Glo-1 expression in response to MGO exposure. We suggest that ST-I4C can be used not only as an easily accessible natural anti-inflammatory material but also as an ingredient for functional foods and pharmaceutical agents related to NAFLD.

## 4. Materials and Methods 

### 4.1. Preparation of Indole-4-carboxaldehyde from S. thunbergii (ST-I4C)

ST-I4C was isolated as previously described [22]. Briefly, a methanol extract was partitioned in chloroform. The chloroform layer was fractionated by silica column chromatography with the stepwise elution of the chloroform–methanol mixture (30:1→1:1) to separate active fractions. A combined active fraction was further subjected to a Sephadex LH-20 column saturated with 100% methanol and then purified by reversed-phase high-performance liquid chromatography (HPLC) using a Waters HPLC system (Alliance 2690; Waters Corp., Milford, MA, USA) equipped with a Waters 996 photodiode array detector and C18 column (J’sphere ODS-H80, 250 × 4.6 mm, 4 μm; YMC Co., Kyoto, Japan) by stepwise elution with a methanol–water gradient (ultraviolet absorbance detection wavelength, 296 nm; flow rate, 1 mL/min). The purified compound was identified by comparing its 1H- and 13C-NMR data with those in the literature [60]. The chemical structure of ST-IC4 is presented in Figure 1A, and its purity was >97%. The compound was dissolved in dimethyl sulfoxide (DMSO) and used in experiments in which the final concentration of DMSO in the culture medium was adjusted to <0.01%. 

### 4.2. Cell Culture

The HepG2 human hepatocyte cell line was obtained from the American Type Culture Collection (ATCC, Manassas, VA, USA). The cells were cultured in high glucose DMEM with L-glutamine (WelGENE, Gyeongsangbuk-do, Korea) supplemented with 10% FBS (WelGENE), 100 U/mL penicillin, and 100 μg/mL streptomycin (WelGENE) and were maintained in a humidified incubator with 5% CO_2_. In all experiments except that to assess viability, cells were cultured in 100 μM ST-I4C for 2 h before the addition of 0.25 mM methylglyoxal (MGO) (Sigma, St. Louis, MO, USA). Dimethyl sulfoxide (DMSO, Duchefa Biochemie, Haarlem, Netherlands) was used for the ST-I4C vehicle, and PBS (WelGENE) used for the MGO vehicle.

### 4.3. Cell Viability 

Cell viability was estimated using a D-PlusTM CCK assay kit (Dongin LS, Seoul, Korea) that measures water-soluble tetrazolium salt. For the assay, HepG2 cells (1 × 10^4^ cells/well) were seeded on 96-well plates. After 16 h, the cells were treated with ST-I4C. The cells were treated with different concentrations of ST-I4C (50, 100, and 200 μM) for 24 h at 37 °C to assess toxicity. The CCK-8 solution was then added to the wells for a total reaction volume of 110 μL. After 1 h of the incubation, the absorbance was measured at a wavelength of 450 nm. The optical density of the formazan generated in the control cells was considered to represent 100% viability.

### 4.4. Quantitative Real-Time PCR

RT-qPCR was performed following the method of Cha et al. [61]. Total RNA was extracted from cells using RNAiso Plus (Takara Bio Inc., Kusatsusi, Japan), and cDNA was prepared using the PrimeScript™ cDNA synthesis kit (Takara Bio Inc.) according to the manufacturer’s instructions. cDNA samples were analyzed using the SYBR^®^ Premix Ex Taq™, ROX plus (Takara Bio Inc.) on Bio-Rad cyclers (Bio-Rad, Hercules, CA, USA). Gene expression was normalized to that of the endogenous housekeeping control gene cyclophilin, which was not influenced by ST-I4C or MGO. Relative expression was calculated for each gene using the ΔΔCT (where CT is the threshold cycle) method. Statistical analysis of the PCR data was based on triplicate samples. The primer sequences are presented in Table 1.

### 4.5. Quantification of Cytokines

HepG2 cells (2 × 10^4^ cells/well) were plated into 48-well plates for cytokine secretion measurements. Briefly, the cells were incubated with or without (control) MGO for 24 h at 37 °C. The supernatants were collected, and released cytokine was measured using an enzyme-linked immunosorbent assay (ELISA) kit according to the manufacturer’s protocol (Abcam, Cambridge, UK). Cytokine content was normalized to protein level, which was determined using a DC™ protein assay kit (Bio-Rad, Hercules, CA, USA).

### 4.6. Western Blotting

HepG2 cells (2 × 10^5^ cells/well) were seeded on 6-well plates, and to assess Glo-1 expression, the cells were incubated with the indicated concentration of ST-IC4 for 24 h; to assess the anti-inflammatory effect, the cells were treated with/without vehicle (control) or 100 µM ST-IC4 for 2 h and then further incubated with or without 0.25 mM MGO for 4 h. For whole cell lysis, the cells were lysed using RIPA buffer (GenDEPOT, USA) and a protease inhibitor cocktail (GenDEPOT) for 30 min on ice, and the lysates were centrifuged at 12,000 rpm for 30 min at 4 °C. To obtain cytosolic and nucleic fractions, the cells were lysed using 1% Triton X-100-PBS and a protease inhibitor cocktail for 20 min on ice. The lysates were centrifuged at 12,000 rpm for 30 min at 4 °C. The supernatant used as the cytosolic fraction, and the pellet was used as the nuclear fraction for western blotting. The protein concentrations were measured using a BCA assay kit (Thermo Fisher scientific, Waltham, MA, USA). The lysates were separated by SDS-PAGE and transferred to PVDF membranes (Millipore, Burlington, MA, USA). Membranes were incubated with 5% skim milk for 1 h at room temperature and then incubated with primary antibodies overnight at 4 °C. After they were washed extensively, the membranes were incubated with the horseradish peroxidase-conjugated secondary antibody (Jackson Immunoresearch, West Grove, PA, USA). The signal was detected using WESTSAVE (Ab Frontier, Seoul, Korea) and an enhanced chemiluminescence system. ImageJ software was used to quantify the band intensity of the western blots. The primary antibodies used were anti-Glo-1 (SC-101537, Santa Cruz Biotechnology, Dallas, TX, USA), anti-p65 (SC-372, Santa Cruz Biotechnology), anti-GAPDH (ab-8245, Abcam, Cambridge, UK), anti-β-actin (SC-47778, Santa Cruz Biotechnology), and anti-lamin B (SC-6216, Santa Cruz Biotechnology).

### 4.7. Determination of AGEs

HepG2 cells (1 × 10^6^ cells/well) were seeded on 100 mm plates, and the cells were incubated with vehicle (control) or 100 µM ST-I4C for 1 h and then further incubated with or without 1 mM MGO for 24 h. The cells were incubated overnight in a chloroform and methanol (2:1 v/v) mixture followed by homogenization in 0.1 N NaOH and centrifugation at 16,000 rpm for 15 min at 4 °C. The supernatant was analyzed for AGE content at an excitation/emission wavelength of 370/440 nm against a 0.1 N NaOH blank on a spectrofluorometer (Victor 3, Molecular Devices, San Jose, CA, USA). A 0.1 mg/mL BSA (bovine serum albumin, Sigma, St. Louis, MO, USA) preparation in 0.1 N NaOH was used as a reference (arbitrary units/mg protein). 

### 4.8. Confocal Microscopy

HepG2 cells (2 × 10^4^ cells/well) cultured on coverslips were incubated with 50 or 100 µM ST-I4C for 24 h. The coverslips were washed twice with PBS and fixed in 4% paraformaldehyde for 15 min at room temperature. The fixed cells were then washed with PBS, blocked with PBS containing 1% BSA and 0.1% Triton X-100 for 30 min at room temperature, and incubated overnight with anti-Glo-1 (Santa Cruz Biotechnology) at 4 °C. Cells were then stained with the fluorescence-conjugated secondary antibody (Life Technologies, Carlsbad, CA, USA) for 2 h, mounted with Vectashield (Vector Laboratories, Burlingame, CA, USA), and observed under a confocal microscope (Zeiss, Oberkochen, Germany). To evaluate Glo-1, four random fields were selected in each experiment, and 20–30 cells were imaged in each field. The fluorescence intensity was measured using ImageJ software.

### 4.9. Transfection 

siRNA for both human Glo-1 and control were purchased from Bioneer (Deajeon, yusung-gu, Korea). HepG2 cells (1.2 × 10^5^ cells/well) cultured in 60 mm cell culture dishes; 16 h later, cells were transfected using lipofectamine RNAiMax (Invitrogen, Carlsbad, CA, USA) according to the manufacturer’s instructions. After 48 h of transfection, the cells were incubated with the vehicle (control) or 100 µM ST-I4C for 24 h and then subjected to Western blotting.

### 4.10. Statistical Analysis 

All measurements were carried out in triplicate, and all values are presented as the mean ± S.E. The results were subjected to analysis of variance with Tukey tests to analyze the differences. Values of *p* < 0.05 were considered significant.

## Figures and Tables

**Table 1:** Primer sequences.

Gene Name		Sequence 5ʹ-3ʹ
Glo-1	Forward	ATG CGA CCC AGA GTT ACC AC
	Reverse	CCA GGC CTT TCA TTT TAC CA
RAGE	Forward	GTG GGG ACA TGT GTG TCA GAG GGA A
	Reverse	TGA GGA GAG GGC TGG GCA GGG ACT
TNF-α	Forward	GAG ATC AAT CGG CCC GAC TA
	Reverse	ACA GGG CAA TGA TCC CAA AG
IFN-γ	Forward	GTA GCG GAT AAT GGA ACT CTT TTC TT
	Reverse	AAT TTG GCT CTG CAT TAT TTT TCT G
Cyclophilin	Forward	TGC CAT CGC CAA GGA GTA G
	Reverse	TGC ACA GAC GGT CAC TCA AA

Glo-1: glyoxalase 1, RAGE: receptor for AGE, TNF-α: tumor necrosis factor-α, IFN-γ: interferon-γ.

## Supplementary Materials

The following are available online at https://www.mdpi.com/1660-3397/17/9/486/s1, Figure S1: MGO was not induced IL-1β and IL-6 cytokines mRNA expression.
