# Supplementary material for: Indole-4-carboxaldehyde Isolated from Seaweed, Sargassum thunbergii, Attenuates Methylglyoxal-Induced Hepatic Inflammation
Source: Mar Drugs. 2019 Aug 21;17(9):486. doi: 10.3390/md17090486 (PMC6780312; doi:10.3390/md17090486)
Supplement: Supplementary file 1 [file marinedrugs-17-00486-s001.pdf]

Supplementary date

Indole-4-carboxaldehyde Isolated from Seaweed, *Sargassum thunbergii*, Attenuates Methylglyoxal-Induced Hepatic Inflammation

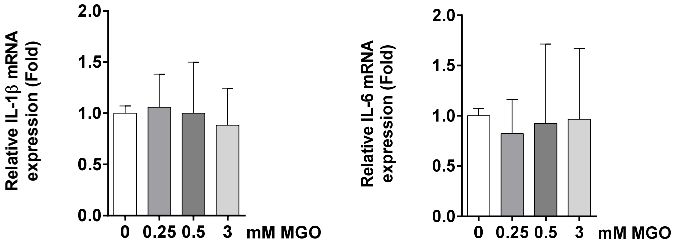

Supplementary figure 1. MGO was not induced IL-1β and IL-6 cytokines mRNA expression.
